# Infection of red blood cells by SARS-CoV-2: new evidence

**DOI:** 10.31744/einstein_journal/2021CE6285

**Published:** 2021-04-08

**Authors:** Uzzam Ahmed Khawaja, Erfan Shamsoddin, Lorenzo Ferro Desideri, Marcos Roberto Tovani-Palone

**Affiliations:** 1 Jinnah Medical and Dental College Department of Medicine Karachi Pakistan Department of Medicine, Jinnah Medical and Dental College, Karachi, Pakistan.; 2 National Institute for Medical Research Development Tehran Iran National Institute for Medical Research Development, Tehran, Iran.; 3 University of Genoa Department of Neurosciences, Rehabilitation, Ophthalmology, Genetics, Maternal and Child Health GenoaGE Italy Department of Neurosciences, Rehabilitation, Ophthalmology, Genetics, Maternal and Child Health, University of Genoa, Genoa, GE, Italy.; 4 Universidade de São Paulo Faculdade de Medicina de Ribeirão Preto Ribeirão PretoSP Brazil Faculdade de Medicina de Ribeirão Preto, Universidade de São Paulo, Ribeirão Preto, SP, Brazil.

Dear Editor,

Severe acute respiratory syndrome coronavirus 2 (SARS-CoV-2) corresponds to a new coronavirus subtype, which was first identified in December 2019 in Wuhan City, Hubei Province, China. This virus is the causative agent of the current pandemic, whose associated disease was named coronavirus disease 2019 (COVID-19) by the World Health Organization (WHO). Thenceforward, SARS-CoV-2 has spread quickly across almost all countries and it has caused various economic and social impacts, affecting globally the health systems and the routine of the population. Despite this, so far, no specific drugs have been made available for intervention and treatment of COVID-19 patients.^(^^1^^)^

The COVID-19 clinical features can include various symptoms, such as fever, myalgia, dry cough, dyspnea, fatigue, radiological evidence of ground-glass lung opacities compatible with atypical pneumonia, diarrhea, and neurological manifestations. Furthermore, its severity is extremely variable, and for this reason, this disease can be classified as asymptomatic, mild, moderate, or severe.^(^^1^^,^^2^^)^

Although several studies have been conducted on the COVID-19 pathophysiology, there are still many concerns about this subject matter. In this regard, a lingering question deals with the mechanism of SARS-CoV-2 infection of red blood cells (RBC). Thus, here we discuss briefly this topic.

First of all, it is necessary to consider that the infection of RBC by SARS-CoV-2 can play a pivotal role in the severity of hypoxemia in COVID-19 patients, further damaging the lower respiratory tract via the angiotensin-converting enzyme 2 (ACE2) receptors. This may be due to the possibility that the virus passes through the alveolar membrane, finding its way to infect RBC, which simultaneously causes a rapid drop in blood oxygen levels.^(^^3^^)^

Moreover, different infectious pathogens, in turn, can invade RBC. This can occur directly and may lead to intravascular hemolysis, or indirectly result in hemolysis or accelerated RBC clearance from the bloodstream via the splenic and hepatic reticuloendothelial phagocytes.^(^^3^^)^

A recent study by Cosic et al., using their own biophysical resonant recognition model (RRM), brings new evidence on how SARS-CoV-2 can infect RBC, making it possible to better understand the COVID-19 pathophysiology.^(^^4^^)^ According to their findings, there might be a likely interaction between the RBC Band 3 surface protein and the S1 spike protein in the SARS-CoV-2 virus, representing a possible entrance of SARS-CoV-2 into RBC. This hypothesis is of paramount significance as the integrity of the RBC Band 3 protein is mandatory for the RBC physiology. Alterations to this mechanism can lead to significant damages to the RBC functions, such as oxygen delivery.^(^^5^^)^ In this case, hypoxia should ensue (as detected in severe COVID-19 cases) due to this interaction, which may decrease the oxygen transport through RBC.

Complementary to this, increased levels of glycolytic intermediates, accompanied by the oxidation and fragmentation of ankyrin, spectrin beta, and the N-terminal cytosolic domain of Band 3 (AE1) have been observed in COVID-19 patients. Significantly altered lipid metabolism was also noted, in particular, involving short- and medium-chain saturated fatty acids, acyl-carnitines, and sphingolipids.^(^^3^^)^ These findings are of great importance given that recent evidence has delineated the significant positive association of RBC distribution width with mortality risk in hospitalized COVID-19 patients.^(^^6^^)^

On the other hand, a previous study referred that RBC Band 3 protein acts as an entry point for merozoites from plasmodium falciparum into the RBC. However, based on the RRM model, it was found that the characteristic frequency for this parasite in merozoite form and the RBC Band 3 protein is different from the RRM frequency for SARS-CoV-2 and the RBC Band 3 protein, thereby suggesting the existence of different mechanisms for these two interactions.^(^^4^^)^ This understanding is undoubtedly a key point for future studies that may establish more effective therapeutic protocols for the treatment of COVID-19 severe cases.

In summary, in light of the hypothesis stated above, we expect that effective improvements will be provided for the management of severe cases of the disease, and especially with regard to devising therapeutic regimens that help to maintain the RBC functions.^(^^4^^)^ In this context, it should be clear to all health professionals that COVID-19 is a systemic disease and therefore may affect other organs besides the lungs.^(^^1^^,^^2^^)^ In this way, it is imperative that advances regarding the pathophysiology of the disease would be taken into account in clinical settings (either private or public facilities) and hospital practice.
