# Supplementary material for: Infection of red blood cells by SARS-CoV-2: new evidence
Source: Einstein (Sao Paulo). 2021 Apr 8;19:eCE6285. doi: 10.31744/einstein_journal/2021CE6285 (PMC8029641; doi:10.31744/einstein_journal/2021CE6285)
Supplement: Translation

# Translation: Infecção de hemácias por SARS-CoV-2: novas evidências

**Authors:** Uzzam Ahmed Khawaja, Erfan Shamsoddin, Lorenzo Ferro Desideri, Marcos Roberto Tovani-Palone

**Affiliations:** 1 Karachi Pakistan Department of Medicine, Jinnah Medical and Dental College, Karachi, Pakistan.; 2 Tehran Iran National Institute for Medical Research Development, Tehran, Iran.; 3 GenoaGE Italy Department of Neurosciences, Rehabilitation, Ophthalmology, Genetics, Maternal and Child Health, University of Genoa, Genoa, GE, Italy.; 8 Ribeirão PretoSP Brasil Faculdade de Medicina de Ribeirão Preto, Universidade de São Paulo, Ribeirão Preto, SP, Brasil.

Caro Editor,

O coronavírus da síndrome respiratória aguda grave 2 (SARS-CoV-2) corres­ponde a um novo subtipo de coronavírus, que foi identificado pela primeira vez em dezembro de 2019, na cidade de Wuhan, localizada na província de Hubei, na China. Esse vírus é o agente causador da atual pandemia, cuja doença associada foi denominada pela Organização Mundial da Saúde (OMS) como doença pelo coronavírus 2019 (COVID-19). Desde então, o SARS-CoV-2 tem se espalhado rapidamente em diversos países, causando diversos problemas econômicos e sociais, o que tem acarretado prejuízos para os sistemas de saúde e a rotina da população em um nível global. No entanto, até o momento, nenhum medicamento específico está disponível para intervenção e tratamento de pacientes com a doença.^(^^1^^)^

As características clínicas da COVID-19 incluem diversos sintomas, como febre, mialgia, tosse seca, dispneia, fadiga, evidência radiológica de opacidades de vidro fosco nos pulmões compatíveis com pneumonia atípica, diarreia e manifestações neurológicas. Contudo, a gravidade da COVID-19 é extremamente variável. Por tal razão, essa doença pode ser classificada como assintomática, leve, moderada ou grave.^(^^1^^,^^2^^)^

Apesar de diversos estudos terem sido conduzidos sobre a fisiopatologia da COVID-19, ainda existem muitas dúvidas sobre o assunto. Nesse sentido, um questionamento que permanece refere-se ao mecanismo de infecção das hemácias por SARS-CoV-2. Aqui, discutiremos brevemente esse tópico.

Primeiro, é necessário considerar que a infecção das hemácias por SARS-CoV-2 pode ter papel determinante na gravidade da hipoxemia em pacientes com COVID-19, resultando em maiores danos ao trato respiratório inferior, por meio da ação da enzima conversora da angiotensina 2 (ECA2). Isso deve ocorrer pela possibilidade de o vírus ultrapassar a membrana alveolar, criando uma via para infecção das hemácias, o que, simultaneamente, deve levar a uma rápida redução no nível de oxigênio sanguíneo.^(^^3^^)^

Além disso, diferentes microrganismos infecciosos podem invadir as hemácias. Isso pode ocorrer tanto diretamente, ocasionando hemólise intravascular, quanto indiretamente, resultando em hemólise ou destruição acelerada das hemácias na corrente sanguínea, por meio de fagócitos esplênicos e do sistema reticuloendotelial hepático.^(^^3^^)^

Estudo recente realizado por Cosic et al., utilizando seu próprio modelo de reconhecimento ressonante (MRR) biofísico, traz novas evidências de como o SARS-CoV-2 pode infectar as hemácias, o que possibilita compreender melhor a fisiopatologia da COVID-19.^(^^4^^)^ Suas descobertas sugerem uma provável interação envol­vendo o vírus SARS-CoV-2 e as células vermelhas do sangue, mais especificamente entre a proteína de superfície de banda 3 das hemácias e a proteína *spike* S1 presente no vírus, o que representa uma possível via de entrada do SARS-CoV-2 para o interior dessas células. Essa hipótese é de suma importância, dado que a integridade da proteína de superfície de banda 3 das hemácias é imprescindível para a fisiologia das células vermelhas do sangue. Alterações nesse mecanismo podem levar a danos significativos nas funções eritro­citárias, como para o fornecimento de oxigênio.^(^^5^^)^ Nesse caso, pode ocorrer hipóxia, como detectado em casos graves de COVID-19, devido a esse tipo de interação, a qual pode reduzir o transporte de oxigênio mediado pelas células vermelhas do sangue.

Complementarmente a isso, níveis aumentados de intermediários glicolíticos, em conjunto com a oxidação e fragmentação de anquirina, betaespectrina e do domínio citosólico N-terminal da banda 3 (AE1) de hemácias, têm sido observados em pacientes com COVID-19. Alterações significativas no metabolismo lipídico também foram notadas, em particular envolvendo ácidos graxos saturados de cadeias curta e média, acilcarnitinas e esfingolipídios.^(^^3^^)^ Tais achados são de grande importância, posto que evidências recentes têm demonstrado associação positiva significante entre a amplitude de distribuição das hemácias e o risco de mortalidade em pacientes hospitalizados com COVID-19.^(^^6^^)^

Por outro lado, estudo prévio reportou que a proteína de banda 3 das hemácias atua como ponto de entrada para merozoítos de *plasmodium falciparum* invadirem as células vermelhas do sangue. Porém, com base no modelo MRR, encontrou-se que a frequência característica para esse parasita na forma de merozoíta e a proteína de banda 3 das hemácias é diferente da frequência de MRR para SARS-CoV-2 e essa mesma proteína, sugerindo, portanto, a existência de mecanismos diferentes para essas duas interações.^(^^4^^)^ Essa compreensão é, sem dúvida, um ponto-chave para estudos futuros que possam estabelecer protocolos terapêuticos mais efetivos voltados ao tratamento de casos graves de COVID-19.

Em suma, à luz das hipóteses apresentadas, esperamos que melhorias efetivas sejam implementadas para o tratamento de casos graves da doença, especialmente com relação à elaboração de esquemas terapêuticos que auxiliem na manutenção das funções das hemá­cias.^(^^4^^)^ Nesse contexto, deve ficar claro a todos os profissionais de saúde que a COVID-19 é uma doença sistêmica e, portanto, pode acometer outros órgãos além dos pulmões.^(^^1^^,^^2^^)^ Dessa forma, é crucial que os avanços quanto à fisiopatologia da doença sejam levados em consideração tanto em ambientes clínicos (quer sejam em serviços privados ou públicos), quanto na prática hospitalar.
